# Spatiotemporal dynamics of Aurora B-PLK1-MCAK signaling axis orchestrates kinetochore bi-orientation and faithful chromosome segregation

**DOI:** 10.1038/srep12204

**Published:** 2015-07-24

**Authors:** Hengyi Shao, Yuejia Huang, Liangyu Zhang, Kai Yuan, Youjun Chu, Zhen Dou, Changjiang Jin, Minerva Garcia-Barrio, Xing Liu, Xuebiao Yao

**Affiliations:** 1 Anhui Key Laboratory of Cellular Dynamics and Chemical Biology, University of Science & Technology of China, Hefei 230027, China; 2 Anhui-MSM Joint Research Group for Cellular Dynamics, Hefei National Laboratory for Physical Sciences at Nanoscale, Hefei 230027, China; 3 Department of Physiology, Morehouse School of Medicine, Atlanta, GA 30310, USA

## Abstract

Chromosome segregation in mitosis is orchestrated by the dynamic interactions between the kinetochore and spindle microtubules. The microtubule depolymerase mitotic centromere-associated kinesin (MCAK) is a key regulator for an accurate kinetochore-microtubule attachment. However, the regulatory mechanism underlying precise MCAK depolymerase activity control during mitosis remains elusive. Here, we describe a novel pathway involving an Aurora B-PLK1 axis for regulation of MCAK activity in mitosis. Aurora B phosphorylates PLK1 on Thr210 to activate its kinase activity at the kinetochores during mitosis. Aurora B-orchestrated PLK1 kinase activity was examined in real-time mitosis using a fluorescence resonance energy transfer-based reporter and quantitative analysis of native PLK1 substrate phosphorylation. Active PLK1, in turn, phosphorylates MCAK at Ser715 which promotes its microtubule depolymerase activity essential for faithful chromosome segregation. Importantly, inhibition of PLK1 kinase activity or expression of a non-phosphorylatable MCAK mutant prevents correct kinetochore-microtubule attachment, resulting in abnormal anaphase with chromosome bridges. We reason that the Aurora B-PLK1 signaling at the kinetochore orchestrates MCAK activity, which is essential for timely correction of aberrant kinetochore attachment to ensure accurate chromosome segregation during mitosis.

During cell division, accurate chromosome segregation requires dynamic interactions between kinetochores and spindle microtubules (MTs), which results in accurate chromosome bi-orientation^1^,^2^,^3^,^4^. Kinesin-13 family is a key regulator required for spindle microtubule dynamics in mitosis^5^,^6^. MCAK is the best-characterized microtubule depolymerase in kinesin-13 family^7^,^8^. As a microtubule-end stimulated ATPase^9^,^10^, MCAK promotes MT catastrophe at both ends *in vitro* and orchestrates spindle microtubule dynamics *in vivo*^11^,^12^,^13^. The MT depolymerization activity of MCAK relies both on its binding affinity to and turnover rate at the MT ends^9^,^14^,^15^.

During mitosis, MCAK corrects erroneous kinetochore attachment via destabilizing improperly attached microtubules^16^. The accurate regulation of MCAK depolymerase activity is proposed to be essential for genomic stability as depletion of MCAK results in aberrantly attached kinetochores and missegregated chromosomes^16^,^17^,^18^. However, the precise mechanism of action underlying MCAK activity control was less characterized. During the last decade, phospho-regulation of MCAK was widely investigated. In early mitosis, cyclin-dependent kinase 1 (CDK1), Aurora A, p21-activated kinase (PAK1), and probably Ca^2+^/Calmodulin-dependent protein kinase IIγ (CaMKIIγ) phosphorylate MCAK to ensure bipolar spindle assembly^19^,^20^,^21^,^22^. In prometaphase, the N-terminus of MCAK is phosphorylated by Aurora B at centromeres, which is required for proper kinetochore-microtubule (KT-MT) attachment^23^,^24^,^25^,^26^. Recently, our previous study revealed that phosphorylation of MCAK C-terminus by Polo-like kinase 1 (PLK1) stimulates MCAK activity in cells and might be essential for faithful chromosome segregation^27^. However, the spatiotemporal dynamics of MCAK phosphorylation by PLK1 during mitosis and the precise mechanism of action underlying PLK1-elicited MCAK phosphorylation remain elusive.

PLK1 is a critical serine/threonine kinase that regulates multiple stages of mitosis^28^,^29^. It was reported that Aurora A-Bora phosphorylates PLK1 on Thr210 and thereby activates PLK1 at centrosomes^30^,^31^. However, how PLK1 is activated and maintained in an active state at different organelles in mitosis remains less characterized. Aurora B shares a high sequence identity with Aurora A^39^, yet it performs different functions depending on its distinct subcellular localization^32^. Since the T-loop activation site of PLK1 lies in a consensus motif for both Aurora A and Aurora B, an outstanding question is whether Aurora B is also involved in activation of PLK1. Our previous study identified that Aurora B kinase phosphorylates PLK1 *in vitro*^33^, which was verified in *Drosophila*^34^. However, the downstream signaling cascade that relays Aurora B-PLK1 activation at the kinetochore was unexplored.

Here, we demonstrate that Aurora B phosphorylates PLK1 to maintain PLK1 activity at the kinetochore for accurate kinetochore bi-orientation and chromosome segregation in mitosis via phospho-regulation of MCAK activity. Unlike the previously reported Aurora B-MCAK pathway that functions in early mitosis, this newly characterized Aurora B-PLK1-MCAK signaling axis ensures error-free chromosome segregation during metaphase-anaphase transition.

## Results

### Phosphorylation of MCAK on Ser715 enhances its binding affinity to microtubules

**Figure 1:**
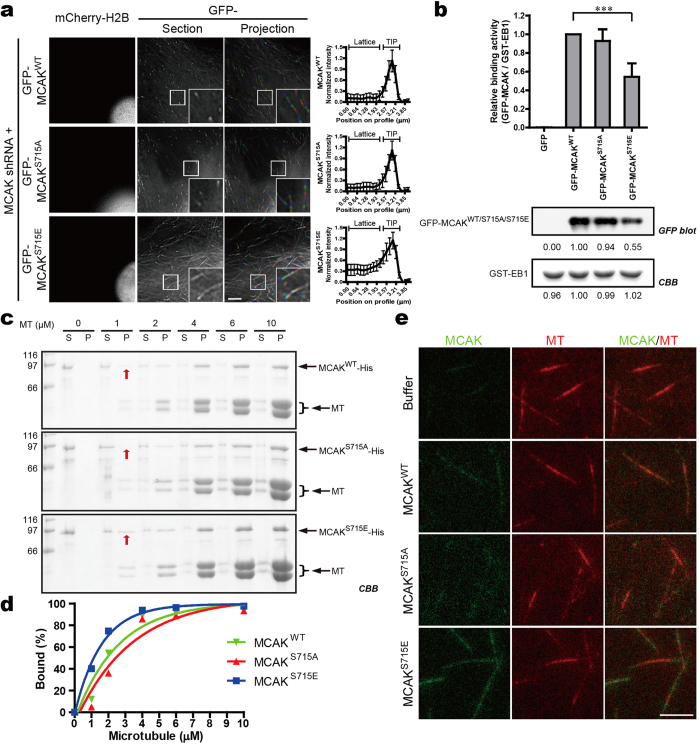
Phosphorylation of MCAK on Ser715 improves its MT-binding affinity. (**a**) MT plus-end location of GFP-MCAK^WT^ or its phosphorylation site mutants in HeLa cells depleted of endogenous MCAK. Time-lapse imaging was performed at 3 sec intervals. Left column is the control for MCAK shRNA transfection as indicated by mCherry-H2B. The second column shows the single frame from the time-lapse images as grayscale. The third column displays projection of 6 consecutive frames, respectively shown as the sequence of red, green and blue. Fluorescence profile of MCAK^WT^ or its mutants along MT plus-end is shown on the *right*. Data are presented as means ± SD derived from at least 60 MT ends for each condition. (**b**) EB1-binding activity of GFP-MCAK^WT^ and its mutants. GST-EB1 was used as an affinity matrix to isolate the indicated GFP fusions from 293T cell extracts. Quantitative analysis of the relative binding affinity by anti-GFP blotting and Coomassie Brilliant Blue (CBB) staining indicated that EB1 bound less to MCAK^S715E^. ***P < 0.001, Student’s *t*-test. For full blots, see Supplementary Fig. S1e. (**c**) MT cosedimentation analysis of MCAK^WT^ or its mutants (1 μM) with increasing concentrations of Taxol-stabilized MTs. Equal volumes of supernatant (S) and pellet (P) were run on SDS-PAGE gels and stained by CBB. (**d**) Graphs showing the MT-binding curves of MCAK^WT^, MCAK^S715A^ and MCAK^S715E^. Data represent the average amounts from two independent experiments. (**e**) Imaging the binding affinity of MCAK^WT^ or its mutants for MTs *in vitro*. Rhodamine-labelled GMPCPP-MTs were immobilized to the surface of coverslips and GTP-MTs were then polymerized by addition of tubulins and GTP. Equal concentration of GFP-His-tagged MCAK^WT^ or its mutants (4 nM) were separately introduced into the chambers and their binding activities for MTs were then observed by total internal reflection fluorescence (TIRF) microscope. Scale bars, 5 μm (all image panels).

Our previous study demonstrated that PLK1 phosphorylates six sites (Ser592, Ser595, Ser621, Ser632, Ser633 and Ser715) within C-terminus of MCAK^27^. To delineate the respective contribution of the aforementioned sites in chromosome segregation, we generated a series of single-site mutants of MCAK and transfected them into HeLa cells. To avoid the interference of endogenous MCAK, an shRNA targeting to the 3′-UTR of MCAK was constructed and employed. In MCAK shRNA positively-transfected cells, MCAK protein was largely suppressed (Supplementary Fig. S1a). Transient transfection to express exogenous MCAK^WT^ and its mutants gave a two-fold expression of MCAK fusion proteins without interference by the shRNA (Supplementary Fig. S1b). More importantly, we found a distinct subcellular location for MCAK when phosphorylated in Ser715. Unlike GFP-MCAK^S715A^ (the nonphosphorylatable mutant in which Ser715 was replaced by alanine) that accumulated at the distal ends of microtubules (MTs), MCAK^S715E^ (the phosphomimetic mutant in which Ser715 was replaced by glutamic acid) shows a long tail following the “comet”-like structures at MT plus ends in living cells (Fig. 1a). These specific phenotypes were also observed in fixed interphase cells and mitotic cells with EB1 staining as a MT plus-end marker (Supplementary Fig. S1 c, d). As MCAK is an EB proteins-dependent MT plus-end tracking protein^6^,^35^,^36^,^37^, we also assessed whether phosphorylation on Ser715 altered the EB1-binding activity of MCAK. To this end, GST-EB1 was used as the affinity matrix to isolate GFP-tagged MCAK^WT^, MCAK^S715A^ or MCAK^S715E^. As shown in Fig. 1b and Supplementary Fig. S1e, MCAK^S715E^ exhibited a reduced binding activity to EB1.

We next determined whether phosphorylation of Ser715 modulates the binding affinity of MCAK to MTs *in vitro*. In this co-sedimentation assay, MTs were stabilized with Taxol to minimize the destabilization of MTs. Consistent with a previous report^38^, MCAK binds to MTs in a dose-dependent manner. Quantification of MCAK fractions in supernatants and pellets showed that MCAK^S715E^ bound to MTs with a relatively higher affinity (Fig. 1c,d), especially in the case of lower concentration of MTs (Supplementary Fig. S1f). To further verify these results, the binding affinity of MCAK to GMP- or GTP-stabilized MTs was also estimated. Pre-assembled GMPCPP-MTs were firstly immobilized on the coverslips. The mixtures, containing equal concentration of GFP-His-tagged MCAK^WT^ or its mutants (Supplementary Fig. S1g), rhodamine-tubulins and GTPs, were subsequently added into the reaction chamber to initiate the polymerization of GTP-MTs. Using total internal reflection fluorescence (TIRF) microscope, MCAK^S715E^ was found to exhibit stronger fluorescent signals at both GMPCPP-MTs and GTP-MTs (Fig. 1e). Thus, we conclude that phosphorylation of MCAK on Ser715 improves its MT-binding affinity.

### Phosphorylation of MCAK on Ser715 stimulates its MT depolymerase activity

**Figure 2:**
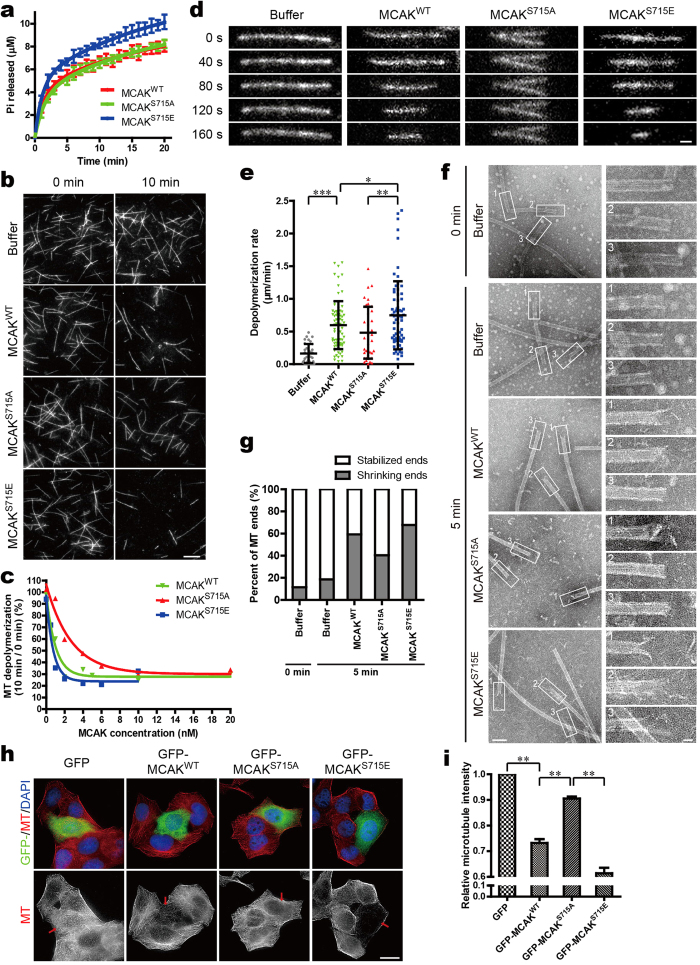
Phosphorylation of MCAK on Ser715 stimulates its MT depolymerization activity. (**a**) Pi release displayed over time in reactions containing 1 mM ATP and 320 nM MCAK^WT^ or its mutants. The data of three independent experiments was fitted to two-phase exponential curves. (**b**) Representative images of MTs incubated with 4 nM MCAK^WT^ or its mutants. Rhodamine-labelled GMPCPP-MTs (1 μM) were incubated with FLAG-MCAK^WT^ or its mutants for 10 min at 25 °C. MTs were then examined by TIRF microscope at the indicated time. Scale bar, 10 μm. (**c**) Plot of MT depolymerization against MCAK concentration. Ratio of MT length at 10 min (post-reaction) over that at 0 min (pre-reaction) was used to reflect MT depolymerization. (**d**) Real-time images of MT depolymerization in the presence of MCAK^WT^ or its mutants. Rhodamine-labelled GMPCPP-stabilized MTs were immobilized as described in Fig. 1e. FLAG-MCAK (2 nM) was then added to initialize MT depolymerization. Scale bar, 1 μm. (**e**) Statistical analysis of the average rate of MT depolymerization during a 5 minutes interval (**d**). Data are presented as means ± SD. At least 40 MTs were measured for each condition. *P < 0.05, **P < 0.01, ***P < 0.001, Student’s *t*-test. (**f**) Negative stained transmission electron micrographs showing the microtubules in the presence of MCAK^WT^ or its mutants. GMPCPP-MTs were incubated with 2 nM MCAK for 5 min at 25 °C. Insets show details of MT ends. Scale bars, 100 nm and 20 nm (enlarged images). (**g**) Statistical analyses of different structures of MT ends seen in (**f**). The blunt ends were considered to be stabilized, while the pared ends were seen as shrinking ends (see also Supplementary Table S2). Data are derived from approximately 60 MT ends for each condition. (**h**) Images of MT density in HeLa cells overexpressing GFP-MCAK^WT^ or its mutants. At 48 hr post-transfection, cells were fixed with methanol and stained with anti-α-tubulin antibody (red) and DAPI (blue). Scale bar, 20 μm. (**i**) Statistical analysis of relative MT intensity in (**h**). Data are presented as means ± SE and derived from at least 60 cells in three independent experiments. **P < 0.01, Student’s *t*-test.

The increased MT-binding affinity of Ser715-phosphorylated MCAK prompted us to check the functional significance of this site on MCAK depolymerase activity. As the ATPase activity of MCAK is necessary for MT depolymerization^9^, we first compared the ATPase rates of purified MCAK^WT^ with its mutants by measuring the kinetics of Pi release (Supplementary Fig. S2a). As shown in Fig. 2a, the basal ATPase rate of MCAK^S715E^ was increased compared to MCAK^S715A^, with a fast rate (0.896 ± 0.177 μM per minute) in the initial phase and a slow rate of Pi release (0.052 ± 0.026 μM per minute) in the following steady-state phase (see also Supplementary Table S1). Thus, Ser715-phosphorylated MCAK exhibited a higher rate of ATPase activity, implying an enhanced depolymerase activity.

To further examine whether phosphorylation of Ser715 regulates the MT depolymerase activity of MCAK, we performed MT depolymerization assay *in vitro*. Purified recombinant MCAK^WT^ and its mutants were incubated with GMPCPP-stabilized MTs, respectively, in the presence of ATP (Supplementary Fig. S2b). MT depolymerization was then quantified by comparing the microtubule length from pre- and post-reaction samples. As shown in Fig. 2c, MT depolymerization accelerated as the concentration of MCAK increased. Even though there was no significant difference in depolymerization between MCAK^WT^ and its mutants at saturated concentrations (>10 nM), it was notable that, at a lower concentration of 4 nM, MCAK^S715E^ displayed a higher depolymerase activity than MCAK^S715A^ (Fig. 2b,c). Using time-lapse TIRF microscopy, depolymerization of individual MT was measured in the presence of various MCAK mutants. As shown in Fig. 2d, a rapid depolymerization was detected after infusion of MCAK. Quantitative analysis of the depolymerization rate indicated that the average rate of MT depolymerization caused by MCAK^S715E^ (0.73 μm/min) significantly increased compared with that induced by MCAK^WT^ (0.60 μm/min) or MCAK^S715A^ (0.48 μm/min) (Fig. 2e), suggesting that phosphorylation of MCAK on Ser715 directly stimulates its MT depolymerization activity *in vitro*.

Since MCAK functions at MT ends during the depolymerization process, we then used Transmission Electron Microscopy (TEM) to get more details about the MT ends in the presence of different MCAK mutants. Pre-polymerized GMPCPP-MTs mixed with MCAK or buffer alone were allowed to react for 5 min before imaging collection. Micrographs acquired by TEM showed that there were more shrinking MT ends in the case of MCAK^S715E^ (67.7%) than that of MCAK^S715A^ (40.4%) (Fig. 2f,g), consistent with the enhanced depolymerase activity of MCAK^S715E^.

We also investigated the depolymerase activities of different MCAK mutants *in vivo* by measuring the α-tubulin immunofluorescence intensity in HeLa cells. The various MCAK proteins were expressed at a comparable level in cells (Supplementary Fig. S2c). Notably, the relative MT intensity in MCAK^WT^-transfected cells was 26.8% lower than that in GFP-transfected cells, consistent with our previous study^27^. By contrast, the relative MT intensity in cells expressing MCAK^S715E^ was significantly lower than that of MCAK^S715A^–expressing cells (Fig. 2h,i; **P < 0.01). Thus, we conclude that phosphorylation of MCAK on Ser715 significantly improves its MT depolymerization activity.

### Phosphorylation of MCAK on Ser715 is spatiotemporally controlled in mitosis

**Figure 3:**
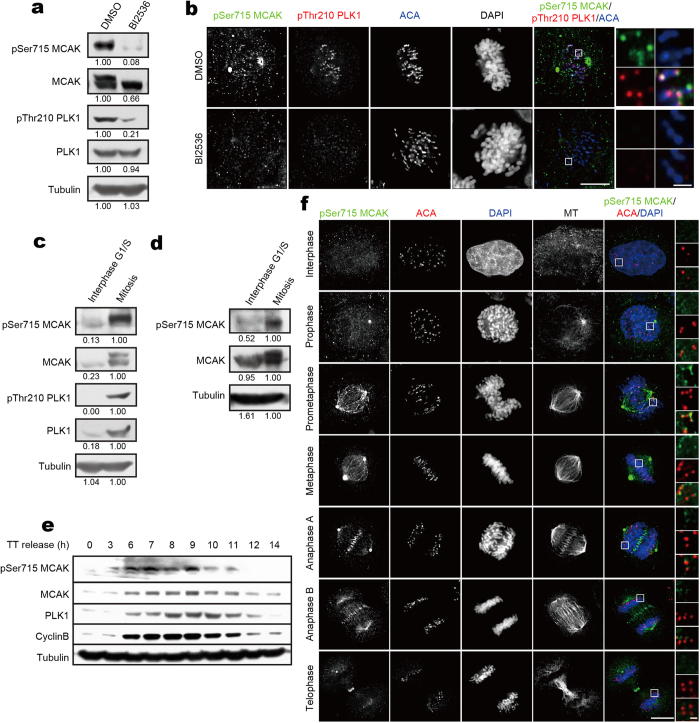
Dynamic phosphorylation of MCAK on Ser715 in cell cycle. (**a**) Nocodazole-arrested HeLa cells were treated with BI2536 or DMSO for another 1 hr, followed by Western blot analysis with antibodies against pSer715-MCAK, MCAK, pThr210-PLK1, PLK1 and α-tubulin, respectively. (**b**) Images of mitotic HeLa cells treated with BI2536 or DMSO. Cell were fixed and stained with anti-pSer715-MCAK antibody (green), anti-pThr210-PLK1 antibody (red), ACA (blue) and DAPI. Insets on the *right* show single focal planes of the boxed regions. Scale bars, 10 μm and 1 μm (enlarged images). (**c**,**d**) HeLa cells were synchronized to G_1_/S boundary by a double thymidine block or prometaphase by Nocodazole treatment. Cells were then harvested and analyzed by Western blotting with the indicated antibodies. Tubulin and MCAK served as a loading control respectively in (**c**) and (**d**). (**e**) Double thymidine treated HeLa cells were released into fresh medium at indicated time. The harvested cells were then analyzed by Western blotting with antibodies against pSer715-MCAK, MCAK, PLK1, CyclinB and α-tubulin (loading control). (**f**) Images of HeLa cells stained with anti-pSer715-MCAK antibody (green), ACA (red), DAPI (blue) and anti-α-tubulin antibody. Representative kinetechores are enlarged and shown on the *right*. Scale bars, 10 μm and 1 μm (enlarged images).

The functional significance of MCAK modification on Ser715 prompted us to further investigate the spatiotemporal dynamics of Ser715 phosphoralytion in cells. Therefore, we generated a phospho-specific antibody against the peptide region around phospho-Ser715 of MCAK, and tested the specificity of this antibody. pSer715-MCAK antibody recognized a specific band and showed a staining on kinetochores and central spindles, while depletion of MCAK by siRNA nearly abolished this signal (Supplementary Fig. S3a,b), demonstrating that pSer715 antibody is specific to MCAK. In addition, the phospho-specific signal of pSer715-MCAK antibody was determined by inhibiting the activity of PLK1 in mitotic cells with a small molecule inhibitor (BI2536). Inhibition of PLK1 abolished the pSer715-MCAK antibody signal in both immunoblotting and immunofluorescence assays (Fig. 3a,b), suggesting that pSer715 antibody is specific to the phosphorylated MCAK. The specificity of the phospho-Ser715 antibody was also evident by the analyses of the exogenously expressed GFP-MCAK^WT^ but not GFP-MCAK^S715A^ or GFP-MCAK^S715E^ (Supplementary Fig. S3c). Those data demonstrated that Ser715 of MCAK is a bona fide substrate for PLK1 *in vivo*.

To assess the temporal order of MCAK phosphorylation by PLK1, aliquots of synchronized HeLa cells were collected for Western blotting analyses. As shown in Fig. 3c, the level of pSer715-MCAK is very low in interphase cells but promptly rises in mitotic cells. Quantitative Western blotting analyses demonstrate that phosphorylation of MCAK on Ser715 is high in prometaphase cells synchronized by nocodazole treatment (Fig. 3d). To determine the temporal partten of Ser715 phosphorylation throughout cell cycle, HeLa cells collected at sequential time points after release from a double thymidine block were analyzed by Western blotting. Pronounced signals of pSer715-MCAK were observed from mitosis entry to anaphase (marked by Cyclin B), particularly at prometaphase (Fig. 3e).

To further reveal the spatiotemporal distribution of phosphorylated MCAK during cell cycle, HeLa cells were fixed and stained with pSer715 antibody. In interphase, almost no phospho-signal was observed (Fig. 3f). In prometaphase and metaphase, pSer715-MCAK was readily seen at centromeres. During the metaphase-anaphase transition, centromeric pSer715-MCAK became liberated, whereas the central spindle-associated pSer715-MCAK increased. Moreover, the pSer715-MCAK was colocalized with activated-PLK1 (marked by pThr210-PLK1 staining) at the aforementioned subcellular structures throughout mitosis (Supplementary Fig. S3d). Our further examination of the pSer715-MCAK antibody specificity using chemical genetic alles demonstrated that the pSer715 is a function of PLK1 (supplementary Fig. S4). Taken together, these data indicate that phosphorylation of MCAK on Ser715 is catalyzed by PLK1 during mitosis.

### Phosphorylation of MCAK on Ser715 is critical for accurate chromosome segregation

**Figure 4:**
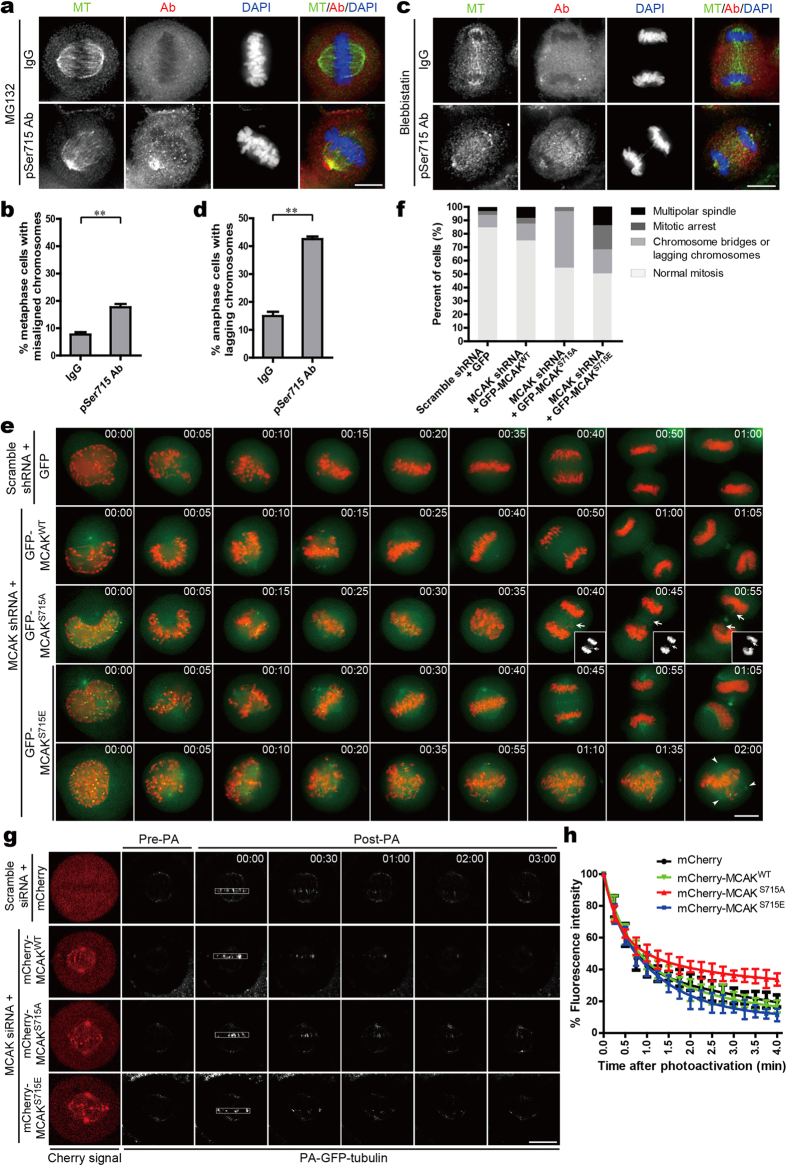
Perturbation of MCAK phosphorylation on Ser715 causes defects in chromosome segregation. (**a**) Images of metaphase cells upon introduction of pSer715-MCAK antibody. Double thymidine-blocked HeLa cells were released and electrotransfected with pSer715-MCAK antibody or control IgG. Cells were then arrested at metaphase by MG132 treatment for 1 hr, followed by fixation and immunofluorescence with a goat anti-rabbit rhodamine-conjugated secondary antibody to highlight the pSer715 antibody in cells. (**b**) Statistical analysis of metaphase cells with misaligned chromosomes seen in (**a**). Data are presented as means ± SD from three independent experiments. **P < 0.01, Student’s *t*-test. (**c**) Images of anaphase cells injected with pSer715-MCAK antibody. HeLa cells were treated as described in (**a**) except that cells were synchronized in anaphase by Blebbistatin (a small molecule inhibitor of Myosin). (**d**) Statistical analysis of anaphase cells with chromosome bridges or lagging chromosomes seen in (**c)**. Data are presented as means ± SD from three independent experiments. **P < 0.01, Student’s *t*-test. (**e**) Live-cell imaging of chromosome movements in HeLa cells co-transfected with GFP-MCAK constructs and mCherry-H2B-marked MCAK shRNA. At 48 hr post-transfection, cells were observed with a DeltaVision microscopy system for two hours. Arrows indicate lagging chromosomes during segregation. Arrowheads indicate multiple spindle poles. Time is annotated as hr:min. (**f**) Quantitative analysis of mitotic phenotypes seen in (**e**). Data are derived from over 30 cells for each category. (**g**) Time-lapse imaging of metaphase spindle dynamics in MCAK^WT^-, MCAK^S715A^- or MCAK^S715E^-expressing cells. HeLa cells depleted of endogenous MCAK by siRNA were co-transfected with PA-GFP-tubulin and mCherry-MCAK. Cells were synchronized in metaphase and imaged before (Pre-PA) and after photoactivation (Post-PA). Time is shown as min:sec. (**h**) Fluorescence intensity over time after photoactivation in (**g**). GFP intensity in the activated region was calculated and normalized to the first time-point after photoactivation. Data are represented as means ± SD and derived from over 10 cells for each condition. Scale bars, 10 μm (in all image panels).

The spatiotemporal distribution pattern of pSer715-MCAK suggests a critical role of PLK1-mediated phosphorylation of MCAK in chromosome movements during mitosis. To directly examine the role of phosphorylated MCAK in chromosome dynamics, aliquots of purified pSer715-MCAK antibodies were introduced into HeLa cells to sequestrate the phosphorylated form of MCAK followed by examination of chromosome positioning. Our preliminary characterization indicated that the electroporated pSer715-MCAK antibodies are located to kinetochore and mitotic spindle (Supplementary Fig. S5). As shown in Fig. 4a,b, introduction of pSer715-MCAK antibody into mitotic cells resulted in a two-fold increase in cells bearing misaligned chromosomes at metaphase (17% in antibody-injected cells versus 8% of control rabbit IgG-injected cells). Moreover, a large number of pSer715-MCAK antibody injected cells exhibited chromosome bridges or lagging chromosomes in anaphase (Fig. 4c,d; 43% of pSer715 antibody-injected cells compared to 15% in control). As suppression of MCAK often leads to anaphase defects^16^,^17^,^18^, the phenotypes seen in pSer715-MCAK antibody-injected cells suggest that pSer715 phosphorylation is essential for mitotic progression and chromosome stabilty in mitosis.

To further investigate the functional requirement of PLK1-mediated phosphorylation of MCAK in mitotic process, the ability of different MCAK mutants to compensate for MCAK depletion in chromosome alignment and separation was recorded by time-lapse video microscopy. As shown in Fig. 4e,f, up to 41.9% MCAK^S715A^-expressing cell exhibited anaphase defects with chromosome bridges or lagging chromosomes, which was a much higher frequency than that in MCAK^WT^ or MCAK^S715E^-expressing cells. Surprisingly, addback of GFP-MCAK^S715E^ resulted in slight increases of cells arrested at prometaphase (17.9%) and cells with multi-polar spindles (13.8%) (Fig. 4e,f). As phosphorylation of MCAK on Ser715 regulates its depolymerase activity (Fig. 2), the mitotic defects in MCAK^S715E^-expressing cells are probably due to the persistent hyperactivity of MCAK^S715E^ and abnormal spindle assembly. On the contrary, reduced activity of non-phosphorylatable MCAK may lead to hyperstability of spindle microtubules and hence chromosome segregation defects. These findings are consistent with the previously reported phenotypes in MCAK-depleted cells^16^,^17^,^18^. Therefore, we conclude that the accurate regulation of MCAK activity by PLK1 phosphorylation on Ser715 is essential for faithful chromosome alignment and segregation during mitosis.

The above results suggested that defects of MCAK Ser715 phosphorylation result in hyper-stabilization of kinetochore microtubules, which may lead to failures in detachment of microtubules from the incorrect pole. To test this hypothesis, spindle microtubule dynamics was evaluated in MCAK^WT^ or its Ser715 mutants addedback to cells following depletion of endogenous MCAK by siRNAs. Time-dependent decrease in fluorescence intensity of photoactivated GFP-tubulin within the activated region was calculated to monitor MT dynamics (Fig. 4g). The double-exponential curve fit suggested that spindle microtubules in MCAK^S715A^-expressing cells are static compared to those of MCAK^WT^-expressing or MCAK^S715E^-expressing cells (Fig. 4h). Thus, our data demonstrate that reduced depolymerase activity of MCAK^S715A^ inhibits spindle microtubule dynamics, which may account for the chromosome missegregation seen in these cells.

### Aurora B is involved in regulation of MCAK Ser715 phosphorylation through activation of PLK1

**Figure 5:**
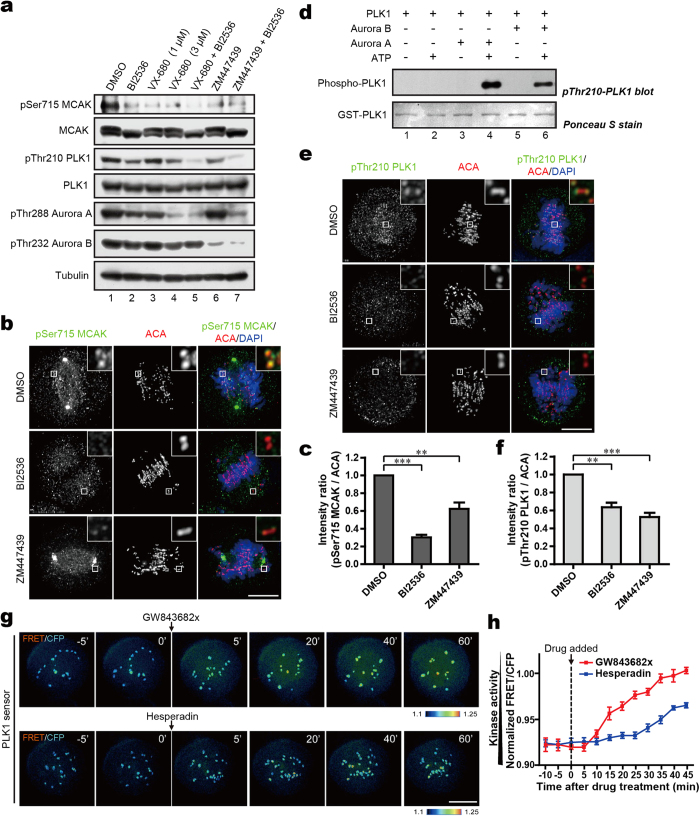
Aurora B is involved in PLK1-dependent phosphorylation of MCAK through activation of PLK1 at the centromeres. (**a**) HeLa cells synchronized to mitosis by Nocodazole were treated respectively with BI2536, VX-680, ZM447439 or DMSO for another 1 hr, followed by Western blot analysis with the indicated antibodies (**b**,**e**) Synchronized mitotic HeLa cells were treated with BI2536, ZM447439 or DMSO for 1 hr. Cells were then fixed and stained with anti-pSer715-MCAK antibody or anti-pThr210-PLK1 antibody (green), ACA (red) and DAPI (blue). The enlargements show the representative centromeres. (**c**,**f**). Statistical analysis of pSer715-MCAK and pThr210-PLK1 immunofluorescence intensity at centromeres in (**b**) and (**e**), respectively. The intensity ratio in DMSO-treated group was normalized to 1. Data are shown as means ± SE and derived from at least 10 cells for each condition. **P < 0.01, ***P < 0.001, Student’s *t*-test. (**d**) Recombinant GST-PLK1 was phosphorylated by Aurora kinases. *In vitro* phosphorylation reactions were performed as described in Methods. GST-PLK1 was visualized by Ponceau S staining and phosphorylation was detected by the antibody against pThr210-PLK1 by Western blotting. (**g**) Color-coded images of HeLa cells expressing a centromere-targeted PLK1 FRET sensor in prometaphase of cells arrested by Taxol treatment. PLK1 inhibitor (GW843682x) or Aurora B inhibitor (Hesperadin) was added at the indicated time point during live-cell imaging. Timestamps relative to drug addition is annotated in min. (**h**) Statistical analysis of the FRET/CFP emission ratio on centromeres at the indicated time (**g**). Data are presented as means ± SE derived from over 100 kinetochores of each category from five different cells. Scale bars, 10 μm (all image panels).

MCAK is under regulation of multiple kinases in mitosis. Since the *Xenopus* Ser719 (xMCAK Ser719) corresponding to human MCAK Ser715 was previously suggested as an Aurora A-phosphorylatable site *in vitro*^20^, it is worth investigating whether other mitotic kinases could also be involved in modulation of MCAK Ser715 phosphorylation in mammalian cells. To this end, cells were treated with a series of kinase inhibitors and the levels of pSer715-MCAK were analyzed. When the kinase activity of PLK1 was effectively inhibited after treatment with BI2536, the majority of pSer715-MCAK signal disappeared (Fig. 5a, lane 2). In addition, reduction of pSer715-MCAK signal was also observed in Aurora kinase inhibitor-treated cells (Fig. 5a, lanes 4, 6), implying the potential role of Aurora kinases in modulating Ser715 phosphorylation. As Aurora A localizes to the centrosomes, the phosphorylation of MCAK on kinetochores was unlikely attributed to Aurora A, though we can not rule out that Aurora A may phosphorylate a small subset of MCAK at centrosomes. To validate whether Aurora B contributes to MCAK Ser715 phosphorylation at kinetochores, the fluorescent stains of pSer715-MCAK were analyzed in cells treated with the inhibitor for PLK1 or Aurora B. At kinetochores, pSer715-MCAK signal was briefly decreased in cells treated with the Aurora B inhibitor, comparing with that in PLK1-inhibited cells (Fig. 5b,c).

Since our *in vitro* phosphorylation assay showed that Aurora B does not directly phosphorylate MCAK at the C-terminus, we reasoned that the brief reduction of pSer715 in Aurora inhibitor-treated cells could be mediated by a kinase downstream from Aurora (Supplementary Fig. S6a). Since Aurora A acts as an upstream kinase responsible for PLK1 activation at the centrosomes via phosphorylation of PLK1 Thr210^30^,^31^, we next assessed whether Thr210 could also be phosphorylated by Aurora B. Indeed, an *in vitro* phosphorylation assay showed that Aurora B directly phosphorylated PLK1 on Thr210 (Fig. 5d, lane 6). The staining of pThr210-PLK1 antibody in cells further strengthened this conclusion as inhibition of Aurora B activity reduced pThr210 signal at the kinetochores (Fig. 5e,f), consistent with previous findings in *Drosophila* cells^34^. Therefore, Aurora B is responsible both for phosphorylation of PLK1 on Thr210 *in vitro* and for maintaining PLK1 activation at the kinetochore in cells.

To monitor the temporal dynamics of PLK1 activity in living cells, we sought to engineer a fluorescence resonance energy transfer (FRET)-based sensor that reports quantitative changes in PLK1 substrate phosphorylation in space and time^40^,^41^. As shown in Supplementary Fig. S6b, changes in intra-molecular FRET between cyan and yellow fluorescent proteins (CFP–YFP) depend on changes in phosphorylation of a PLK1 substrate peptide that is conserved in Myt1^41^. The sensor is specific for PLK1 since it does not respond to other mitotic kinases (Supplementary Fig. S6c), indicating that the measured FRET change in cells is a faithful reporter for PLK1 kinase activity. To validate the sensor response to changes in PLK1 activity in living cells, we first imaged mitotic cells before and after kinase inhibition. As shown in Fig. 5g and h, quantitative analysis of FRET/CFP ratio demonstrated that FRET efficiency increased over time after addition of Aurora B kinase inhibitor, indicating PLK1 activity at kinetochores was briefly reduced after inhibition of Aurora B kinase activity. Thus, we conclude that Aurora B indirectly promotes the phosphorylation of MCAK on Ser715 at the kinetochores through phosphorylation of PLK1 at Thr210 and its ensuing activation.

### A dynamically regulated Aurora B-PLK1-MCAK signaling cascade is required for timely correction of aberrant kinetochore attachment

**Figure 6 f6:**
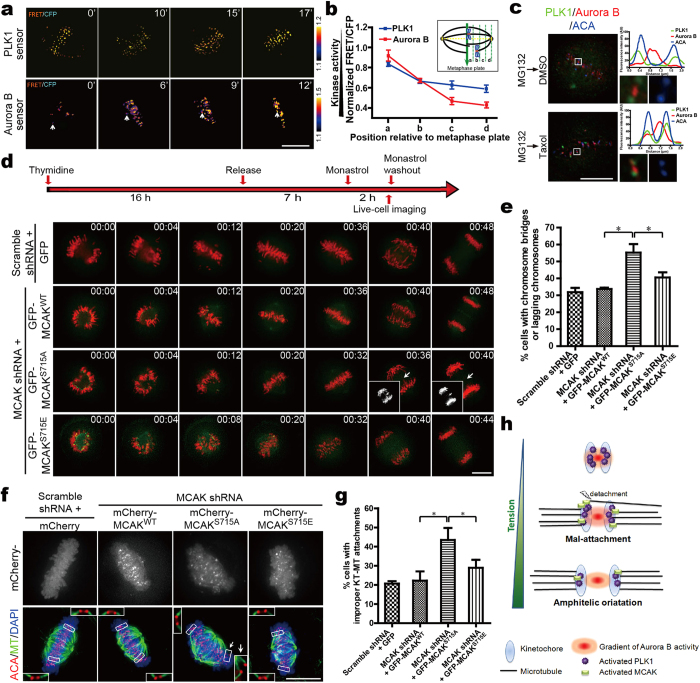
Precise regulation of Aurora B-PLK1-MCAK axis ensures proper kinetochore bi-orientation. (**a**) Color-coded images of HeLa cells expressing PLK1 or Aurora B sensor show chromosome alignment after Syntelin washout. Cells were treated with 1 μM Syntelin for 30 min followed by three washes for subsequent real-time imaging analyses. Timestamps are in minutes. (**b**) Quantitative analysis of FRET/CFP emission ratio on centromeres relative to the metaphase plate. Schematic illustration of centromere position calculation (see also Supplementary Fig. S5a). (**c**) HeLa cells were treated with MG132 for 1 hr to allow for metaphase plate formation. Parallel samples were then treated with 1 μM Taxol or DMSO for 40 min before staining for PLK1 (green), Aurora B (red) and ACA (blue). Insets show individual kinetochore pairs used for line scans. (**d**) Live-cell imaging of chromosome segregation in GFP-MCAK constructs-addback cells. Cells were treated according to the protocol outlined in the upper panel. Arrows indicate lagging chromosomes during segregation. Timestamps in hr:min. (**e**) Quantitative analysis of the defective anaphases seen in (**d**). Data are presented as means ± SD from three independent experiments. *P < 0.05, Student’s *t*-test. (**f**) Cold-stable KT-MT attachment in mCherry-MCAK mutants-addback cells. Cells treated with Monastrol were released into MG132-containing medium for another 1 hr, and then fixed and stained for MTs (green), ACA (red) and DAPI (blue). Arrows indicate erroneous kinetochore attachments. (**g**) Quantitative analysis of cells exhibiting one or more aberrant attachments in various mutant MCAK-expressing cells. Data are presented as means ± SD from three independent experiments. *P < 0.05, Student’s *t*-test. (**h**) Model of accurate regulation of MCAK by Aurora B-PLK1 axis at kinetochore. Aurora B locates closely to PLK1 at prophase, enabling PLK1 to be activated by Aurora B at inner-centromeres (upper row). During the prometaphase-to-metaphase transition, stretch on kinetochore pairs may separate PLK1 and MCAK from Aurora B to outer-kinetochores. The stimulated MCAK activity ensures error correction in KT-MT attachment (middle row). Once the amphitelic attachments are generated, both activities of PLK1 and MCAK are down-regulated, and anaphase begins with chromosomes segregating towards the opposite poles (lower row). Scale bars, 10 μm (all image panels).

To gain further insight into the spatiotemporal pattern and activation of PLK1 by Aurora B, the kinetochore-targeted PLK1 sensor and Aurora B sensor^42^ were utilized to probe their dynamic activities during chromosome alignment. In this assay, a CENP-E inhibitor Syntelin^43^ was used to synchronize cells at prometaphase followed by removal to allow cells to progress into metaphase by correcting chromosome mal-orientation. FRET emission ratios on kinetochores at different positions relative to the metaphase plate were evaluated (Supplementary Fig. S7a) to evaluate the effects of activity changes for the indicated kinases during chromosome congression. As shown in Fig. 6a[Fig f6]b, FRET efficiencies of both PLK1 sensor and Aurora B sensor increased during chromosome congression, indicating that the activities of both PLK1 and Aurora B peaked on unaligned kinetochores, and gradually decreased as the chromosomes were positioning towards the metaphase plate to get bi-orientated. Similarly, staining with anti-pSer715-MCAK and anti-pThr210-PLK1 antibodies revealed higher fluorescence intensity at misaligned kinetochores when compared to that at the aligned kinetochores (Supplementary Fig. S7b,c). These findings suggest that Aurora B may initially phosphorylate and activate PLK1 on unaligned chromosomes, and such regulation may respond to tension generated between sister kinetochores during chromosome alignment.

To further delineate this regulation, we assessed immunostaining of Aurora B and PLK1 at centromeres which are under full tension or reduced tension (Supplementary Fig. S7d). As shown in Fig. 6c, PLK1 partially overlapped with Aurora B at misaligned centromeres under reduced tension in taxol-treated cells. However, the peak of Aurora B localization is largely separated from that of PLK1 due to the tension across the sister kinetochores in MG132-treated cells. Further examination of pSer715-MCAK relative to that of PLK1 or Aurora B revealed that the localization of pSer715-MCAK was super-imposed onto that of PLK1 but delocated from Aurora B under tension (Supplementary Fig. S7e,f). From those complementary studies, we reason that Aurora B initially activates PLK1 at unaligned centromeres by which PLK1 phosphorylates MCAK for optimal error correction via depolymerizing aberrantly attached kinetochore microtubule.

MCAK has been reported to take part in correcting KT-MT malattachments^16^,^17^, phospho-MCAK controlled by “this newly identified” Aurora B-PLK1 axis is likely responsible for error correction of sister kinetochores under tension. As the phenotypes of anaphase defects could reflect the error correction efficiency of MCAK^16^,^17^, we performed live-cell analyses to monitor chromosome segregation in MCAK^WT^, MCAK^S715A^ or MCAK^S715E^-expressing cells. Comparable to the control group, most MCAK^S715E^- and MCAK^WT^-expressing cells could reassemble bipolar spindles and undergo errorless anaphase after release from Monastrol treatment (Fig. 6d,e). However, more than a half of the MCAK^S715A^-expressing cells exhibited apparent anaphase defects with lagging chromosomes or chromosome bridges (Fig. 6d,e, panel 3). As uncorrected merotelic attachments mainly contribute to chromosome misseparation^3^,^44^,^45^, we speculated that the missegregated chromosomes seen in MCAK^S715A^-expressing cells may attribute to its inability to correct improper attachments before anaphase onset. A more detailed examination of KT-MT attachments in different MCAK mutants-addback cells revealed that the percentage of cells with one or more improper attachments dramatically increased in MCAK^S715A^-addback cells, compared to that in MCAK^WT^ or MCAK^S715E^-addback cells (Fig. 6f,g). These findings further confirmed and explained the phenotypes seen in Fig. 6d,e. Therefore, we conclude that the dynamically regulated activity of MCAK orchestrated by the Aurora B-PLK1 axis are essential for proper chromosome bi-orientation and faithful chromosome segregation during mitosis.

## Discussion

During cell division, accurate chromosome segregation is essential for genomic stability. Precisely regulated activity of MCAK depolymerase ensures proper chromosome bi-orientation and faithful chromosome segregation in mitosis. In this study, we identified a novel pathway in regulating MCAK activity at mitotic centromeres and demonstrated its critical role in faithful chromosome segregation. In early mitosis, Aurora B phosphorylates PLK1 on Thr210, triggering its kinase activity at the kinetochores (Fig. 6h, upper row). During prometaphase to metaphase, stretch on kinetochore pairs may cause the spatial separation of PLK1 and MCAK from Aurora B, which results in release of Aurora B-elicited inhibition of MCAK activity. At the same time, Aurora B-mediated PLK1 activation at the kinetochore triggers the MCAK depolymerase activity via phosphorylation of Ser715. Functionally, PLK1-stimulated activity of MCAK allows it to correct aberrant kinetochore attachments by destabilizing kinetochore attachment errors (Fig. 6h, middle row). Proper kinetochore bi-orientation is then established to ensure the accurate anaphase onset and equal segregation of sister chromatids to two daughter cells (Fig. 6h, lower row). Therefore, the dynamic regulation of KT-MT attachment by Aurora B-PLK1-MCAK pathway safeguards chromosome stability during mitosis.

Phosphorylation of MCAK C-terminus on Ser715 promotes its MT depolymerase activity, which could be due mainly to an enhanced MT-binding affinity and ATPase activity (Figs 1 and 2). The high ATPase rate may improve the efficiency of MCAK to travel toward the MT ends and depolymerize tubulin dimer there^10^,^46^. Since MCAK binds to MTs and hydrolyzes ATPs using its neck and motor domain, the increased MT-binding affinity and ATPase activity of MCAK caused by phosphorylation on C-terminus is probably due to a conformational change of neck and/or motor domain. In fact, an intra-molecular regulatory interaction of MCAK was recently reported^27^,^47^. Aurora B phosphorylation of MCAK N-terminus, which inhibits MCAK depolymerase activity, has been reported to open the conformation of MCAK resulting in its dissociation from MTs^47^. Therefore, PLK1 mediated modification of MCAK on Ser715 may modulate its conformational transition as well, as was also suggested by our recent work^27^. It will be of great interest to establish the structural-functional relationships of “closed” and “open” conformations, elicited by PLK1 and Aurora B, respectively.

As all of the previously identified phosphorylations on MCAK exhibit negative regulation on its depolymerase activity^18^, it is hard to evaluate the functional significance of MCAK in mitosis. Recently, another research group reported that among the six PLK1-mediated phosphorylation sites within the MCAK C-terminus^27^, Ser621 phosphorylation regulates MCAK’s stability in mitosis^48^. Unlike Ser621, our studies here demonstrated that Ser715 is the most functionally important site that promotes depolymerase activity of MCAK (Fig. 2). Using a newly developed and phospho-Ser715 specific antibody, we are the first to reveal the spatiotemporal distribution of activated MCAK during mitosis (Fig. 3). The phospho-Ser715-containing MCAK pool was detected at centromeres from prometaphase to anaphase, implying that in addition to metaphase, PLK1-mediated activation of MCAK may also be involved in kinetochore attachment error correction in prometaphase. Besides, pSer715-MCAK signal was also detected on the spindle poles. From metaphase to anaphase, centromeric pSer715-MCAK becomes liberated but never disappears, whereas the central spindle-associated pSer715-MCAK singal increases, suggesting that phosphorylation of MCAK by PLK1 orchestrates chromosome segregation and central spindle dynamics in anaphase. Although we initially observed a portion of pSer715-MCAK signal located to centrosomes (Supplementary Fig. S3b), the phospho-Ser715 labeling is absent from centrosome in RPE1 cells genetically modified expressing Shokat alleles of PLK1^49^, suggesting that majority of the phospho-Ser715 is rather specifically localized to kinetochore. However, we can not rule out the possibility that a small portion of phospho-Ser715-MCAK on centrosomes may also be phosphorylated by PLK1. Further insights into the potential function of PLK1-phosphorylated MCAK on spindle poles and central spindle are warranted.

It has been reported that suppression of MCAK in CHO cells did not cause discernible problems until metaphase^18^. Consistently, introduction of pSer715-MCAK antibody to disturb the function of phospho-Ser715 in HeLa cells did not cause obvious defects in early mitosis either (data not shown). However, a slight increase of metaphase cells with misaligned chromosomes was detected after pSer715-MCAK antibody addition (Fig. 4a,b), which is comparable with the previous reports that about 15% of MCAK-deficient U2OS cells failed to align all chromosomes at the spindle equator after Monastrol removal^17^. Remarkably, as perturbation or depletion of MCAK leads to anaphase defects^16^,^17^,^18^, increased lagging chromosomes and chromosome bridges arised both in cells injected with pSer715 antibody (Fig. 4c,d) and MCAK^S715A^-expressing cells (Figs 4 and 6) indicating that the function of centromeric phospho-Ser715 MCAK was perturbed. Considering the hypoactivity of MCAK^S715A^, the disruption of phosphorylation on Ser715 may lead to hyperstable KT-MT attachments with the consequence of chromosome mis-segregation and chromosomal instability (CIN).

Aurora B was previously reported to inhibit MCAK activity at inner centromeres^23^,^24^,^25^ to ensure initial KT-MT attachments in early prometaphase. Instead of MCAK, another member of the Kinesin-13 family Kif2b and Aurora B-mediated phosphorylation of KMN are supposed to be the primary pathways to correct malattachments of kinetochores to microtubules during mitosis^17^,^50^,^51^. MCAK was proposed to be most active to correct errors in metaphase^17^,^18^. However, prominent staining of pSer715-MCAK at centromeres from early prometaphase to anaphase implied that although most MCAK activity at inner centromeres is inhibited by Aurora B in early prometaphase^23^,^24^,^25^, a small subset of MCAK at outer centromeres may be activated by PLK1, and to some extent, compensate for Kif2b to correct attachment errors. During the prometaphase-to-metaphase transition, MCAK and PLK1 may be pulled away from Aurora B to the outer-centromeres due to the stretch generated between the kinetochore pairs. As a result, the inhibition of MCAK derived from Aurora B may be released, while PLK1-mediated activation of MCAK emerges at outer-centromeres, ensuring timely correction of aberrant attachments. Therefore, although Aurora B blocks MCAK activity at early mitosis for initial spindle assembly, it also recruits MCAK to the centromeres and activates PLK1 to ensure subsequent activation of MCAK to timely correct the improper attachments of kinetochores to microtubules, indicating all-around roles of Aurora B in regulation of MCAK.

Taken together, this newly identified regulatory pathway of MCAK in mitosis demonstrates a critical role of MCAK in errorless chromosome segregation and reveals a novel coordinated mechanism between Aurora B and PLK1 in safeguarding chromosome stability during mitosis. The Aurora B-PLK1-MCAK axis uncovered here provides a unique view of a previously unidentified molecular mechanism that orchestrates aberrant kinetochore attachment correction during mitosis.

## Methods

### Plasmid construction

GFP-tagged full-length MCAK was previously reported^27^. Point mutations within MCAK were generated using a QuikChange Site-Directed Mutagenesis kit (Stratagene). The cDNA was amplified by PCR and subcloned into p3xFLAG-myc-CMV24 (Sigma), modified pcDNA-mCherry and pFastBac1 (Invitrogen) vectors. The design of the PLK1 sensor is based on a PKC sensor^40^: an FHA2 phospho-Thr-binding domain and a substrate peptide (LLLDSTLSINWD from Myt1) were inserted into a CFP/YFP FRET pair. The sensor was generated with CENP-B fusion to target to centromeres. The Aurora B sensor (CENP-B fusion) and PLK1 sensor (Hec1 fusion) used in experiments for Fig. 6a were described in previous reports^41^,^42^. All plasmid constructs were confirmed by sequencing.

### MT co-sedimentation assay

The cosedimentation assays were performed with Taxol-stabilized MTs as described previously^38^. Briefly, equal molar amounts (1 μM) of purified proteins (MCAK^WT^-His, MCAK^S715A^-His or MCAK^S715E^-His) were incubated in BRB80 (80 mM PIPES, pH 6.8, 1 mM MgCl_2_, and 1 mM EGTA) with various concentrations (0–10 μM) of polymerized MTs. All reactions were incubated for 10 min at 25 °C, and subsequently centrifuged for 10 min at 80,000 rpm in a Beckman TLA100 rotor. The supernatants and pellets were separately diluted with equal volumes of SDS-PAGE sample buffer, boiled, and then subjected to electrophoresis. For visualization and quantification of MT-binding, the fractions of MCAK^WT^ or its mutants partitioned to supernatants and pellets were quantified by densitometry of the stained gel using ImageJ software (NIH).

### *In vitro* MT depolymerization assay

MTs were polymerized with 50 μM tubulin plus 1 mM GMPCPP (Jena Bioscience, Germany) as described previously^52^. Purified MCAK^WT^ or its mutants were separately incubated with the reconstituted MTs in BRB80 containing 1 mM ATP and 1 mM DTT for 10 min at 25 °C. The mixtures were then squashed onto coverslips and examined under a total internal reflection fluorescent (TIRF) microscope configured on an ELYRA system (Carl Zeiss).

To immobilize MTs, flow chambers were prepared as previously described^14^. Chambers were coated with 10% monoclonal anti-biotin antibody (Sigma) followed by blocking with 5% Pluronic F-127 (Sigma). MTs containing biotin-tubulin were then allowed to be absorbed to the surface of coverslips. After a brief washing, the reaction mixture (BRB80 supplemented with 50 mM KCl, 5 mM DTT, 1 mM ATP, 0.15% methylcellulose, 0.25 mg/ml k-casein, an oxygen-scavenging system, and MCAK) was introduced into the chamber. The temperature was maintained at 25 °C. Images were collected with a TIRF microscope.

### *In vitro* phosphorylation

The kinase reactions were performed in 40 μl of 1X kinase buffer (25 mM HEPES, pH 7.2, 50 mM NaCl, 2 mM EGTA, 5 mM MgSO_4_, 1 mM DTT, and 0.01% Brij35) containing GST fusion proteins as substrates, purified kinases and 500 μM ATP. Reaction mixtures were incubated at 30 °C for 30 min, then stopped by SDS sample buffer, and separated by SDS-PAGE.

### Antibodies, siRNAs and shRNAs

Anti-MCAK antibody was reported previously^53^. To generate MCAK phospho-Ser715 antibody, a synthetic peptide containing phospho-Ser715 (CMQLEEQA-pS-RQISS) was conjugated to rabbit albumin (Sigma Chemical) and immunized into rabbits as described^54^. The serum was collected by a standard protocol and preabsorbed by nonphosphorylated MCAK peptide (CMQLEEQASRQISS) followed by affinity-purification using CMQLEEQA-pS-RQISS-conjugated sulftone sepharose beads (Sigma Chemical). Other antibodies were obtained from commercial sources: mouse anti-PLK1 monoclonal antibody (Invitrogen); anti-phospho-Thr210 PLK1 antibody (BD Biosciences); anti-Cyclin B antibody (BD Biosciences); mouse anti-EB1 antibody (BD Biosciences); mouse monoclonal anti-GFP antibody (BD Biosciences); anti-α-tubulin antibody DM1A (Sigma-Aldrich).

MCAK siRNA targeting the 3′-UTR of MCAK gene was purchased from Qiagen (SI05040686). The small-hairpin RNA (shRNA) against MCAK was constructed using the same targeting sequences as its siRNA. To get a fluorescence-marked shRNA system, pLKO.1 cloning vector (Addgene) was reconstituted by inserting a sequence encoding mCherry-H2B to generate a plasmid co-expressing target shRNA and mCherry-H2B.

### Immunofluorescence

For visualization of MT plus-end, HeLa cells growing on coverslips were fixed with pre-cooled methanol at −20 °C for 5 min. To examine cold-stable microtubules, HeLa cells after transfection or drug treatment were incubated on ice for 10 min in L-15 medium with 20 mM HEPES, followed by 10 min of fixation at room temperature with 3.7% formaldehyde in PTEM (100 mM PIPES, pH 6.8, 10 mM EGTA, 1 mM MgCl_2_, and 0.2% Trition X-100). In other cases, cells were washed once with pre-warm PHEM (60 mM PIPES, 25 mM HEPES, pH 6.9, 10 mM EGTA, 2 mM MgCl_2_, and 4 M Glycerol), followed by 1 min of permeabilization with PHEM containing 0.1% Trition X-100. The extracted cells were then fixed with 3.7% formaldehyde in PHEM for 5 min. After being washed three times with PBST (0.05% Tween-20 in PBS), cells were blocked with 1% BSA (Sigma) in PBST for 1 hr, then incubated with primary antibodies for 1 hr, followed by secondary antibodies for 1 hr at room temperature. DNA was stained with 4′,6-diamidino-2-phenylindol-dihydrochloride (DAPI, Sigma). Immunofluorescence images were collected under a DeltaVision wide-field deconvolution microscope system (Applied Precision Inc.) with a resolution of 200 nm as previously described^55^. Z-series stacks were obtained at 0.2-μm steps using a 60X/NA 1.4 PlanApochromat objective (Olympus) with 1 × 1 binning. Images were deconvolved using SoftWoRx (Applied Precision Inc.) and processed with ImageJ.

### Live cell imaging

HeLa cells were grown in glass-bottomed culture dish (MatTek, MA). During imaging, cells were maintained in CO_2_-independent medium (GIBCO BRL) containing 10% FBS and 1% glutamine in a sealed chamber heated to 37 °C. Images at single focal plane were acquired by a DeltaVision deconvolution microscope (Applied Precision Inc.) with a 60X/NA 1.4 PlanApochromat objective (Olympus). To trace chromosomes or kinetochores in mitosis, frames were collected at 4- or 5-min intervals. For tracking MT tips, frames were acquired with a 3-sec interval.

### Measurement of tubulin dynamics in live cells using photoactivation analysis

Photoactivation experiments were performed as previously described^56^. Briefly, MCAK siRNA-transfected HeLa cells grown in a glass-bottom culture dish were then co-transfected PA-GFP-tubulin with mCherry-MCAK^WT^ or its mutants, followed by synchronization. Several pulses from a 405 nm diffraction-limited laser on LSM710 NLO (Carl Zeiss; Germany) were used to photoactivate an area within the spindle. Images were acquired every 15 seconds. Fluorescence intensities in the activated region were measured at each time point using ImageJ. Data were averaged for multiple cells and fitted to a double-exponential curve.

### Measurement of Aurora B and PLK1 activity in live cells using FRET reporters

To test the sensor responses to PLK1 kinase activity, 293T cell lysates expressing FRET-based PLK1 sensor were incubated with either different mitotic kinases or the indicated inhibitors. The static fluorescence emission spectra between 450 and 600 nm were then measured on a Shimadzu RF-5301PC spectrofluorophotometer with excitation at 433 nm. Spectra were normalized and illustrated with GraphPad Prism software.

For live-cell imaging with FRET sensors, CFP was excited at 470 nm, while CFP and YFP emissions were acquired simultaneously with a beam splitter (Dual-View, Optical Insights). In this case, CFP_ex_/CFP_em_ (CFP) and CFP_ex_/YFP_em_ (FRET) were measured from all samples. The FRET emission ratio (FRET/CFP) was calculated by dividing CFP_ex_/CFP_em_ (CFP) to CFP_ex_/YFP_em_ (FRET) using SoftWoRx (Applied Precision Inc.). For statistical analyses, individual centromere/kinetochore was defined automatically from confocal image stacks, and FRET emission ratio on each centromere/kinetochore was measured as previously described^42^. The ratios were normalized by dividing by the maximum value of FRET emission ratio for each experiment. According to the design of the FRET-based sensors, a higher FRET emission ratio indicates a lower kinase activity.

To create syntelic attachment, aliquots of HeLa cells were treated with syntelin (1 μM) for 1 hour followed by three washes^42^,^43^.

### Statistical analyses

For quantifying fluorescence intensity, the value of MT intensity in plasmids-transfected cells over that in surrounding non-transfected cells was calculated as described previously^27^. Kinetochore staining signals were measured with ImageJ software, according to the procedures described previously^56^. The mean value in GFP or DMSO group was normalized to 1. Student’s *t* test was used to evaluate the significance of differences between control groups and MCAK-transfected ones. Differences were considered to be statistically significant when *p* was <0.05.

## Additional Information

**How to cite this article**: Shao, H. *et al.* Spatiotemporal dynamics of Aurora B-PLK1-MCAK signaling axis orchestrates kinetochore bi-orientation and faithful chromosome segregation. *Sci. Rep.* **5**, 12204; doi: 10.1038/srep12204 (2015).

## Supplementary Material

Supplementary Information
