# Supplementary material for: Spatiotemporal dynamics of Aurora B-PLK1-MCAK signaling axis orchestrates kinetochore bi-orientation and faithful chromosome segregation
Source: Sci Rep. 2015 Jul 24;5:12204. doi: 10.1038/srep12204 (PMC4513279; doi:10.1038/srep12204)

Supporting Online Material for

Spatiotemporal dynamics of Aurora B-PLK1-MCAK signaling axis orchestrates kinetochore bi-orientation and faithful chromosome segregation

Hengyi Shao, Yuejia Huang, Liangyu Zhang, Kai Yuan, Youjun Chu, Zhen Dou, Changjiang Jin, Minerva Garcia-Barrio, Xing Liu, & Xuebiao Yao

Supplementary Information

Inventory of Supplementary Information:

Supplementary information contains supplementary experimental procedures, 7 Figures and 2 Tables.

Supplementary Figures

Supplementary Figure S1. Characterization of recombinant MCAK and phospho-mimicking mutants relative to EB1-binding and MT-binding

Supplementary Figure S2. Characterization of the recombinant $MCAK^{WT}$ and its mutants in assays related to Figure 2

Supplementary Figure S3. Specificity of pSer715-MCAK antibody and the co-localization of pSer715-MCAK with activated PLK1 in mitotic cells

Supplementary Figure S4. pSer715-MCAK antibody is a function of PLK1 activity

Supplementary Figure S5. Subcellular localization of electroporated pSer715-MCAK antibody

Supplementary Figure S6. Aurora B does not phosphorylate C-terminus of MCAK and the FRET-based PLK1 sensor

Supplementary Figure S7. Distributions of PLK1, Aurora B and pSer715-MCAK on centromeres responding to tension

Supplementary Tables

Supplementary Table S1. Kinetic measurement of ATPase activities of $MCAK^{WT}$ and its mutants

Supplementary Table S2. Percentages of different structures of MT ends

Supplementary Experimental Procedures

Recombinant protein expression and purification

Human MCAK-His and MCAK-GFP-His were expressed in Sf9 cells (BAC-TO-BAC expression system, Invitrogen) and purified with Ni-NTA resin followed by cation-exchange and desalting. FLAG-tagged MCAK was purified from transiently transfected 293T cells using anti-FLAG M2 beads and followed by elution with FLAG-peptide (Sigma).

Recombinant GST-EB1 and GST-MCAK deletion proteins were expressed in Rosetta (DE3) pLysS (Novagen) under 0.5 mM isopropyl 1-thio-β-D-galactopyranoside (IPTG) at 16℃ for 18 hr. GST-tagged PLK1 was expressed in Sf9 cells. These proteins were then purified with glutathione-Sepharose 4B beads (GE Healthcare) in phosphate-buffered saline (PBS) containing protease inhibitor mixture (Sigma) and 0.5% Triton X-100.

ATPase assays

ATPase rates were determined by measuring the liberated phosphate over time. EnzChek Phosphate Assay Kit (Invitrogen) was used to quantify phosphate release by measuring absorbance at 360 nm. The concentration of phosphate in each sample was determined by comparison of phosphate standard curve.

Cell culture and transfection

HeLa and 293T cells (American Type Culture Collection, Manassas, VA) were cultured in DMEM (Invitrogen) containing 10% FBS (Hyclone, Logan, UT) and 1% glutamine at 37℃ with 8% CO_2.

Plasmids, siRNAs or shRNAs were transfected into cells by Lipofectamine 2000 (Invitrogen, Carlsbad, CA) for 36-48 hr according to the manufacturer's manuals.

Introduction of pSer715-MCAK antibody into HeLa cells by electroporation

Double thymidine blocked HeLa cells were released and electrotransfected with pSer715-MCAK antibody or IgG according to the manufacturer's manual (Lonza, Germany). Briefly, HeLa cells (1X 10^6 cells per sample) were harvested by trypsinization and rinsed once with PBS. Supernatant was then removed completely and 5 μg antibody in 100 μl PBS was added to resuspend the cells. The cell/antibody suspension was transferred into certified cuvette and the electrotransfection was carried out using program I-013. The cells were then spreaded on sterile, acid-treated coverslips and cultured for indicated time.

Drug treatments

Drugs used were Nocodazole (100 ng/ml), Monastrol (100 μM), Taxol (1 μM), MG132 (20 μM), Blebbistatin (100 μM), ZM447439 (2.5 μM), Hesperadin (100 nM), BI2536 (33 nM), GW843682x (1 μM) and Syntelin (1 μM). In some cases, cells were released from drug treatments by three washes with PBS, and then grown in fresh medium for indicated time.

Figure legends

Supplementary Figure S1. Characterization of recombinant MCAK and phospho-mimicking mutants relative to EB1-binding and MT-binding

(a) Characterization of MCAK shRNA knockdown efficiency. An shRNA targeting 3'-UTR of MCAK gene was used, co-expressing with mCherry-H2B to visualize the positively-transfected cells, as indicatead by arrows. MCAK stain in shRNA-transfected cells significantly decreased comparing with that in adjacent non-transfected cells. (b) The expression levels of GFP-MCAK^{WT} and its mutants in endogenous MCAK-depleted HeLa cells. Cells were collected at 48 hr post-transfection and analyzed by Western blotting using indicated antibodies. mCherry-H2B and tubulin served as transfection efficiency and loading control. (c,d) MT plus-end location of MCAK^{WT} or its mutants in interphase or mitotic cells depleted of endogenous MCAK. HeLa cells co-transfected with various GFP fusions and MCAK shRNA were fixed with methanol, and then stained with antibodies against EB1 as a MT plus-end marker. MCAK shRNA transfection as indicated by mCherry-H2B. Enlargements of the selected area showed the details of MCAK distribution at MT plus-ends. (e) GST pull-down assays using GST-EB1 or GST alone as affinity matrix to absorb GFP-tagged MCAK proteins from 293T cell lysates. Western blotting with GFP antibody shows the GFP fusions (upper). CBB staining indicates the GST proteins (lower). (f) MT co-sedimentation analysis of recombinant MCAK^{WT} or its mutants (1 μM). The fractions of MCAK in supernatant (S) and pellet (P) were evaluated by SDS-PAGE followed by silver staining. (g) 1 μg proteins of purified MCAK^{WT} and its mutants used in Fig. 1e. GFP-His-tagged MCAK^{WT} or its mutants were purified from insect cells and detected by SDS-PAGE and CBB staining. Scale bars, 10 μm (all image panels).

Supplementary Figure S2. Characterization of the recombinant MCAK^{WT} and its mutants in assays related to Figure 2

(a,b) Purified MCAK^{WT} and its mutants were detected by SDS-PAGE. His-tagged MCAK used in Fig. 2a were purified from insect cells. FLAG-MCAK used in Fig. 2b-g were obtained from 293T cells. (c) The expression levels of GFP-MCAK^{WT} and its mutants in transfected HeLa cells related to Fig. 2f. The fluorescence intensities of GFP fusions were measured, respectively, to indicate the comparable expression level of these proteins in cells. Date are represented as means ± SE from three independent experiments.

Supplementary Figure S3. Specificity of pSer715-MCAK antibody and the co-localization of pSer715-MCAK with activated PLK1 in mitotic cells

(**a,b**) HeLa cells transfected with scramble or MCAK siRNAs were synchronized to mitosis. At 48 hr post-transfection, MCAK and pSer715 signal in mitotic cells were demonstrated by Western blotting (a) and immunofluorescence (b). Enlarged images in (b) indicate representative kinetochores. (c) GFP-MCAK^{WT} or its mutants-transfected HeLa cells were synchronized to mitosis by Nocodazole. Mitotic cells were collected at 40 hr post-transfection and analyzed by Western blotting using indicated antibodies. MCAK, GFP and tubulin blots served as transfection efficiency and loading control. (d) Co-localization of pSer715-MCAK and activated PLK1 in mitotic HeLa cells. Cells were fixed and stained with anti-pSer715-MCAK antibody (green), anti-pThr210-PLK1 antibody (red) and DAPI (blue). Representative kinetechores are enlarged and shown on the *right*. Scale bars, 10 μm (all image panels).

Supplementary Figure S4. pSer715-MCAK antibody is a function of PLK1 activity

To evaluate the specificity of pSer715-MCAK antibody, we employed the chemical genetic alles of PLK1 as previously reported by Burkard et al., (2007). Specifically, $PLK1^{wt}$ and $PLK1^{as}$ cells were incubated for 10 h with 10 μM 3-MB-PP1 or DMSO. Cells were stained with pSer715-MCAK antibody (red), DAPI (blue) and ACA. The boxed enlargements show the representative centrosomes. Scale bar, 10 μm.

Supplementary Figure S5. Subcellular localization of electroporated pSer715-MCAK antibody

Introduction of pSer715-MCAK antibodies into HeLa cells does not cause obvious spindle defects in interphase/early mitosis. Double thymidine blocked HeLa cells were released and electro-transfected with pSer715-MCAK antibody or control IgG. Cells were then fixed and stained with a goat anti-rabbit rhodamine-conjugated secondary antibody (red), anti-α-tubulin antibody (green) and DAPI (blue). Scale bar, 10 μm.

Supplementary Figure 6. Aurora B does not directly phosphorylate C-terminus of MCAK and the FRET-based PLK1 sensor

(a) Purified GST-MCAK$^{1\text{-}258}$, GST-MCAK$^{587\text{-}725/WT}$ and GST-MCAK$^{587\text{-}725/S715A}$ were seperately incubated with Aurora B kinase in an *in vitro* phosphorylation buffer as described in Methods. GST and GST-histone H3^{NT} were used as the negative and positive control, respectively. (b) Schematic illustration of the FRET-based PLK1 sensor. PLK1 sensor is comprised of mCFP, the FHA2 domain of Rad53p, a PLK1 substrate sequence (LLLDS<u>T</u>LSINWD from Myt1) and mYFP, with CENP-B fusion to target it to centromeres in cells. The substrate peptide, when phosphorylated by PLK1, will bind to FHA2 phospho-Thr-binding domain. Such binding leads to segregation of YFP from CFP and consequently decrease of FRET emission ratio. The conformation change of the sensor could be reversed by phosphatases. (c) FRET spectra indicated that the PLK1 sensor specifically responds to the activity change of PLK1. 293T cells expressing PLK1 sensor with various mitotic kinases were lysed and incubated in the presence of ATP. The fluorescence emission spectra were recorded from 450 and 600 nm with excitation at 433 nm by spectrofluorophotometer. FRET emission dramatically decreased only in the presence of PLK1 kinase indicated the sensor is specific for PLK1 activity.

Supplementary Figure 7. Distributions of PLK1, Aurora B and pSer715-MCAK on centromeres responsive to tension

(a) Schematic illustration of centromere position calculation. Cells imaged in experiments for Fig. 6a were co-transfected with sensor plasmid and mCherry-tubulin. The middle of a spindle was defined as the metaphase plate and the half spindle were divided into four zones (respectively labeled with a, b, c and d) to reflect centromere positions relative to the metaphase plate. (b) Images of prometaphase cells stained with anti-pSer715-MCAK or anti-pThr210-PLK1 antibody (green), ACA (red) and DAPI (blue). Enlarged portions of the selected areas indicate stronger pThr210-PLK1 and pSer715-MCAK stainings on misaligned versus aligned kinetochores. (c) Quantification of pThr210-PLK1 or pSer715-MCAK immunofluorescence intensity on aligned vs. misaligned kinetochores. Data are shown as means ± SE and derived from at least 10 cells for each condition. *P<0.05, **P<0.01, Student's t-test. (d) Synchronized cells were parallelly treated as that in Fig. 6c and stained with anti-α-tubulin antibody (green), ACA (red) and DAPI (blue). Insets show the distance between sister kinetochores reduced after Taxol treatment. (e,f) Parallel samples were treated as that in Fig. 6c and stained with anti-pSer715-MCAK antibody (green), anti-PLK1 antibody or anti-Aurora B antibody (red), and ACA (blue). The boxed enlargements show individual kinetochore pairs used for line scans. Scale bars, 10 µm (all image panels).

Supplementary Table S1. Kinetic measurement of ATPase activities of $MCAK^{WT}$ and its mutants

	K_{Fast}	$T_{1/2,Fast}$	K_{Slow}	$T_{1/2,Slow}$
$MCAK^{WT}$	0.766 ± 0.203 μM/min	0.91 min	0.055 ± 0.040 μM/min	12.53 min
$MCAK^{S715A}$	0.692 ± 0.165 μM/min	1.00 min	0.055 ± 0.030 μM/min	12.50 min
$MCAK^{S715E}$	0.896 ± 0.177 μM/min	0.77 min	0.052 ± 0.026 μM/min	13.44 min

Supplementary Table S2. Statistic analyses of different MT end structures in the presence of MCAK and mutant proteins

The models of MT ends		0 min	5 min			
		Buffer	Buffer	MCAK^{WT}	MCAK^{S715A}	MCAK^{S715E}
Stabilized ends	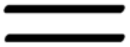	88.5%	81.5%	40.8%	58.5%	32.3%
Shrinking ends	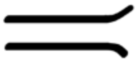	7.7%	8.6%	29.6%	22.6%	32.3%
	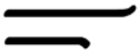	3.8%	7.4%	19.7%	9.4%	23.1%
	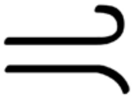	0.0%	2.5%	9.9%	9.4%	12.3%

Supplemental Figure S1

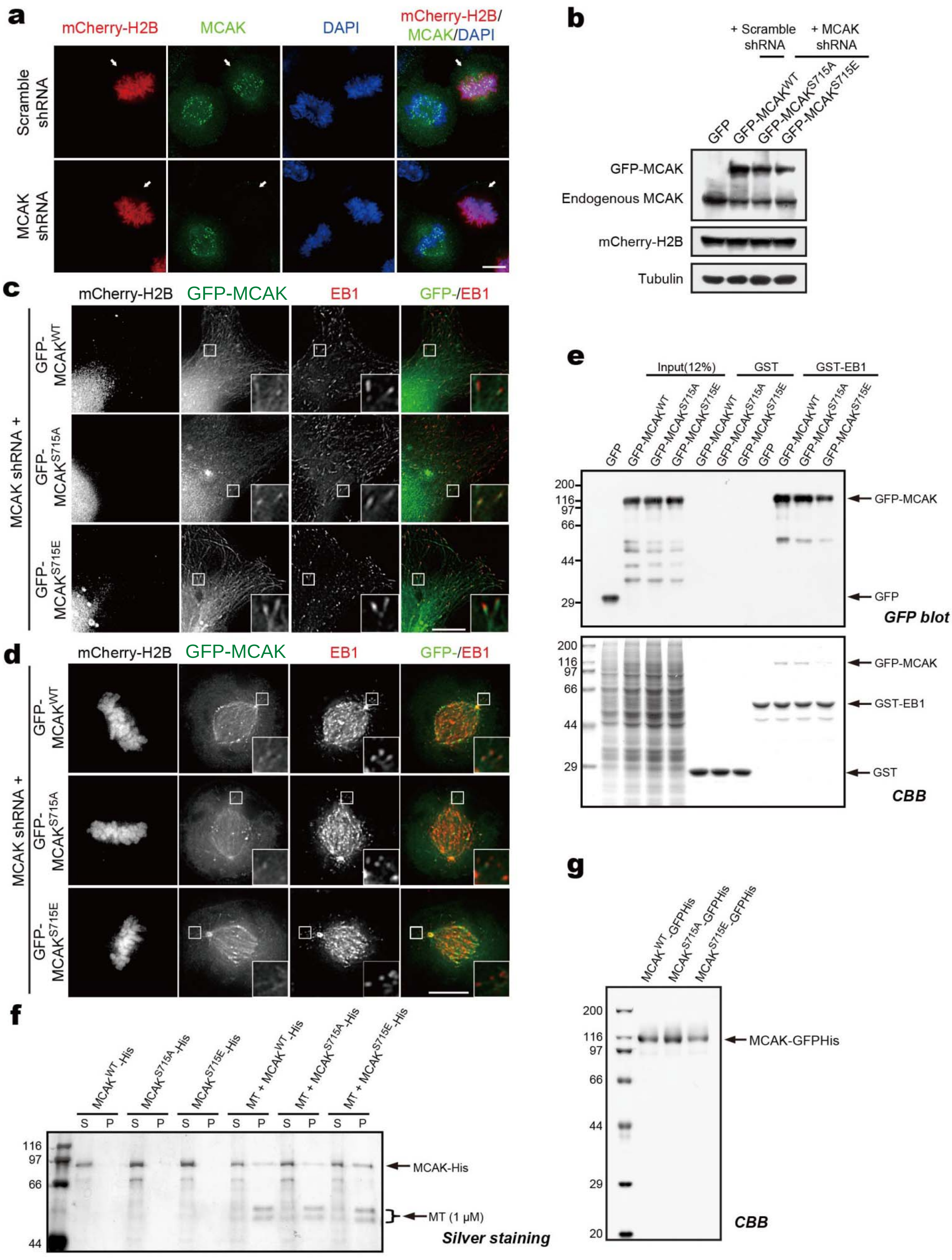

Supplemental Figure S2

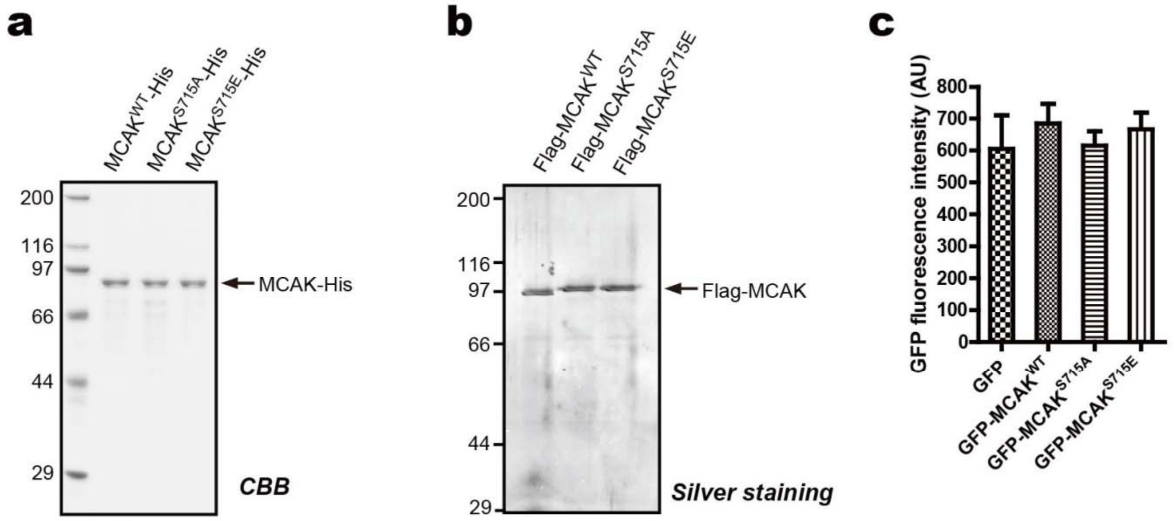

Supplemental Figure S3

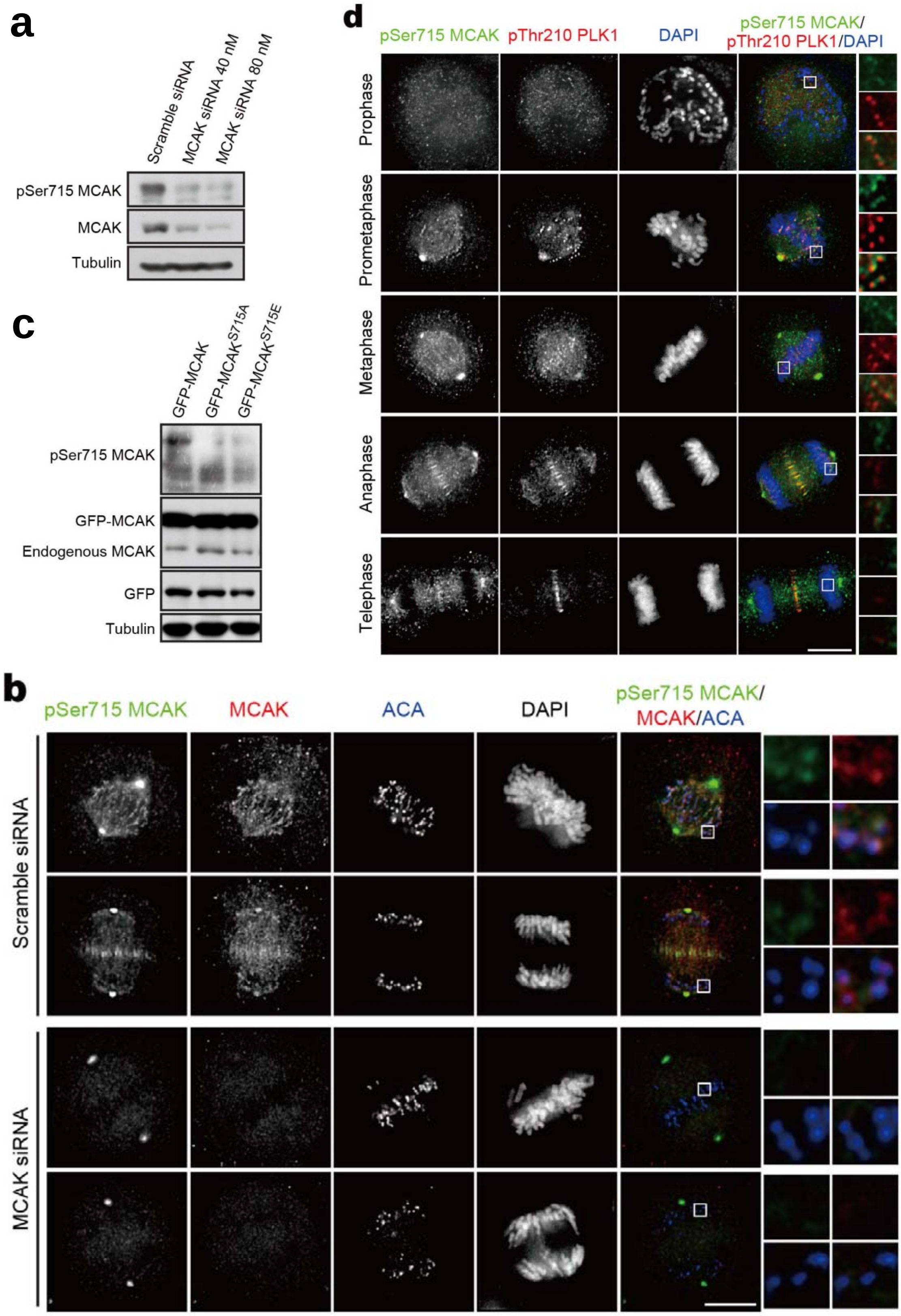

Supplemental Figure S4

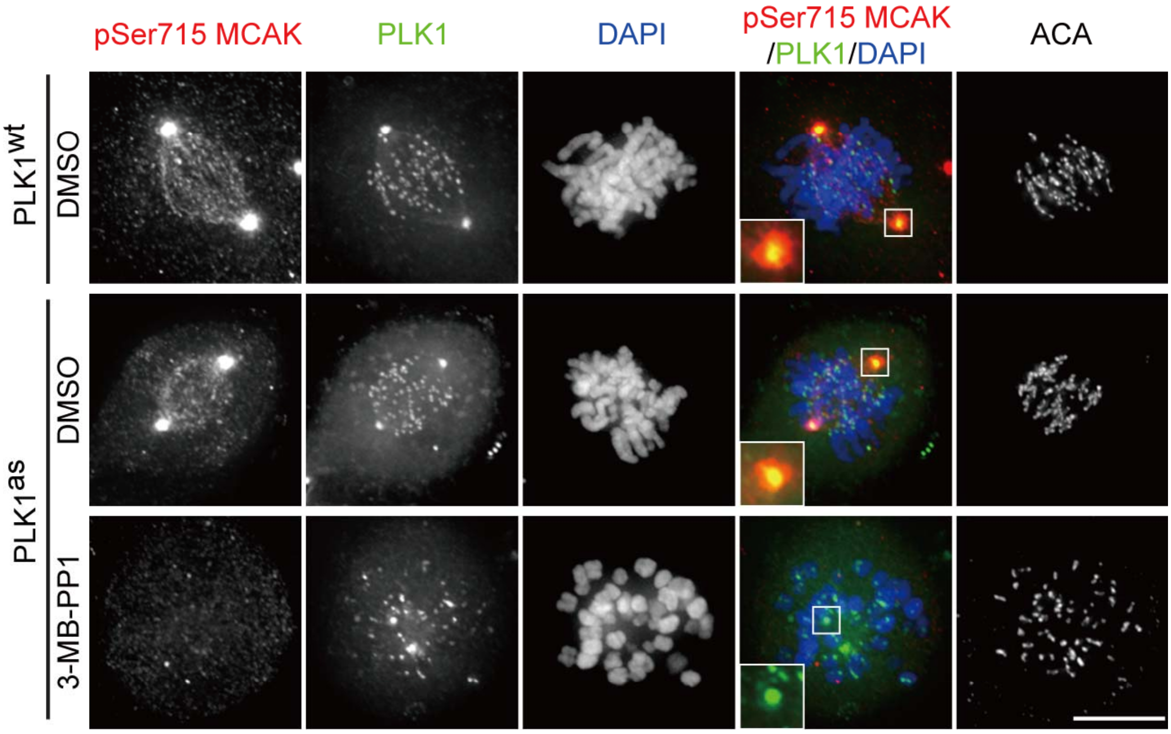

Supplemental Figure S5

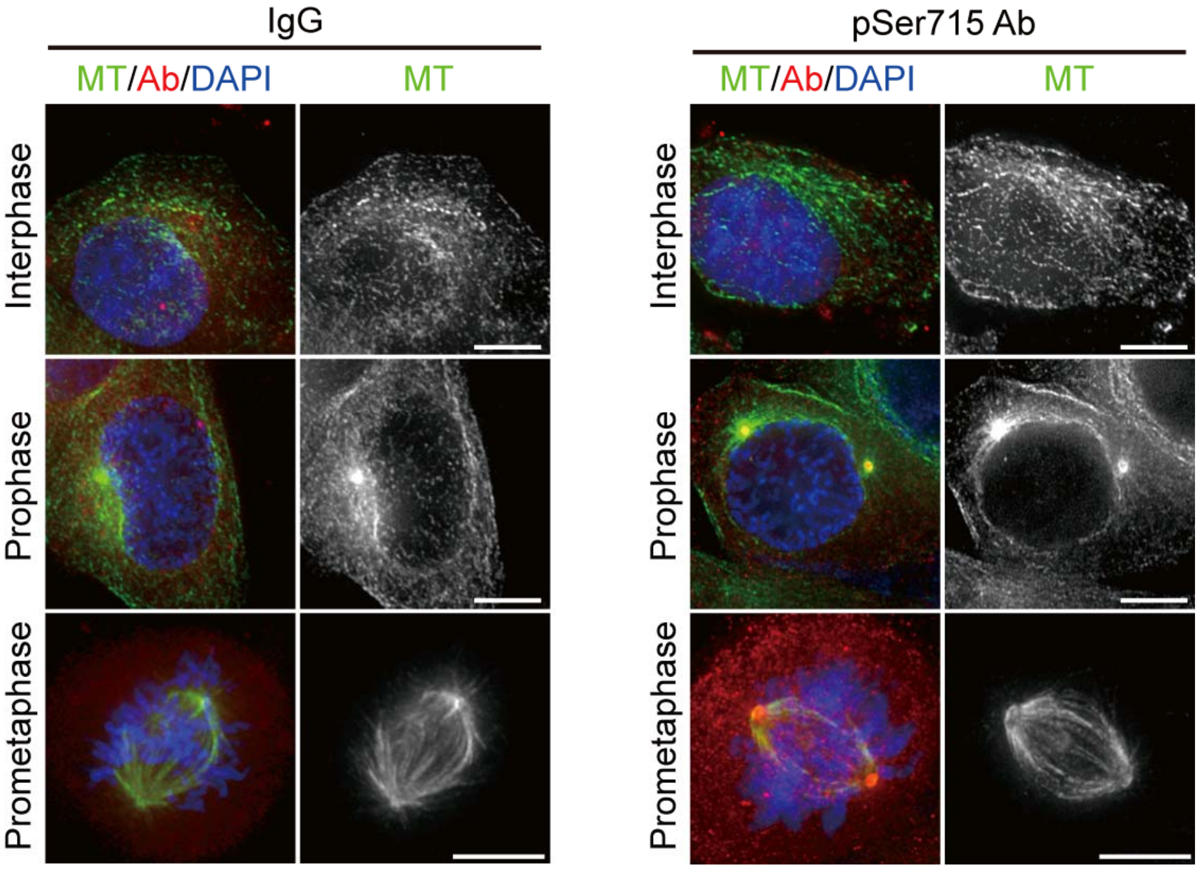

Supplemental Figure S6

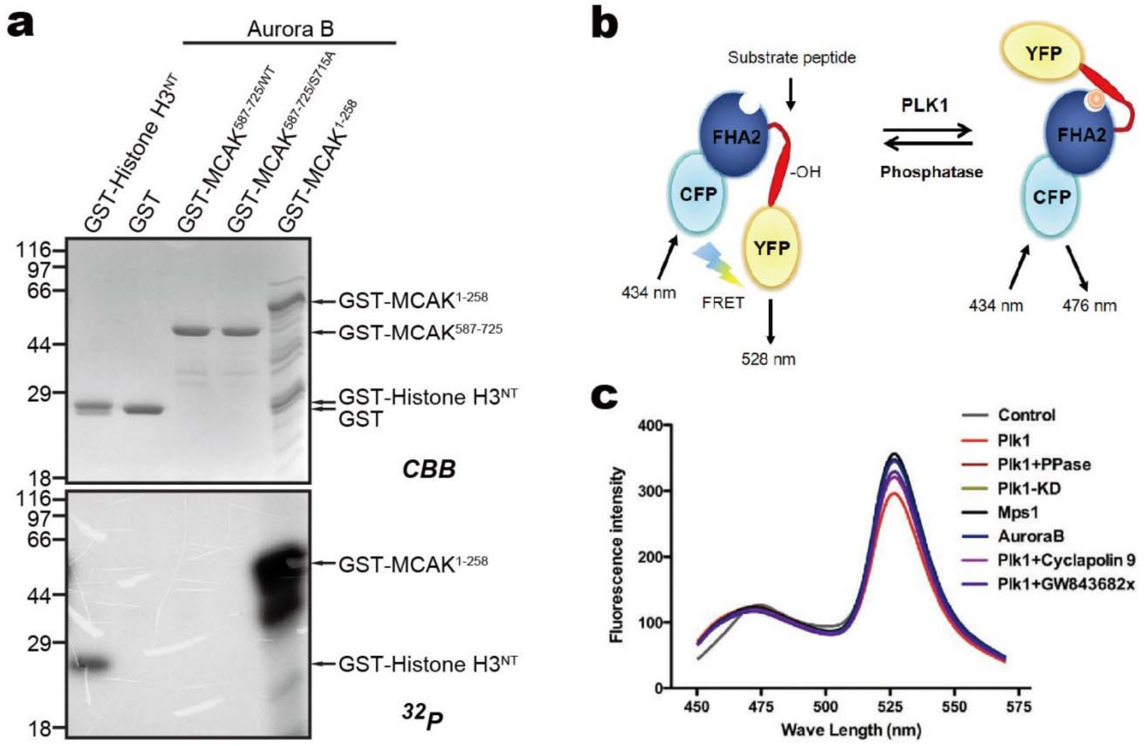

Supplemental Figure S7

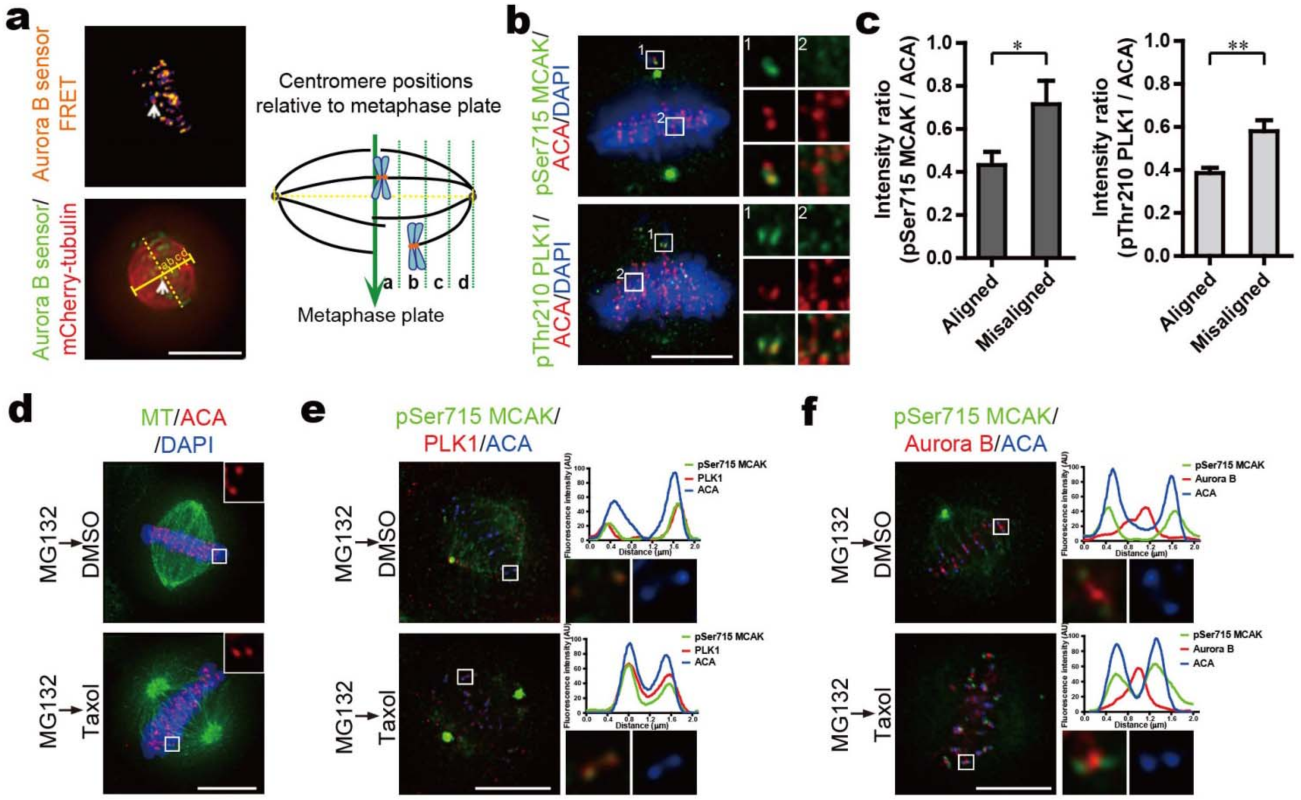

Supplement: Supplementary Information [file srep12204-s1.pdf]
